# Using Minimum Local Distortion to Hide Decision Tree Rules

**DOI:** 10.3390/e21040334

**Published:** 2019-03-28

**Authors:** Georgios Feretzakis, Dimitris Kalles, Vassilios S. Verykios

**Affiliations:** School of Science and Technology, Hellenic Open University, 263 35 Patras, Greece

**Keywords:** decision trees, privacy preserving, hiding rules, entropy, information gain, data sharing

## Abstract

The sharing of data among organizations has become an increasingly common procedure in several areas like banking, electronic commerce, advertising, marketing, health, and insurance sectors. However, any organization will most likely try to keep some patterns hidden once it shares its datasets with others. This article focuses on preserving the privacy of sensitive patterns when inducing decision trees. We propose a heuristic approach that can be used to hide a certain rule which can be inferred from the derivation of a binary decision tree. This hiding method is preferred over other heuristic solutions like output perturbation or cryptographic techniques—which limit the usability of the data—since the raw data itself is readily available for public use. This method can be used to hide decision tree rules with a minimum impact on all other rules derived.

## 1. Introduction

Privacy-protecting data mining [1,2] is a field of research aimed at alleviating the issues arising from the use of data mining about data collection, information or knowledge contained in data collections, and the confidentiality of subjects recorded within them. The first to consider inducing decision-making trees from anonymized data which have been adequately noise-driven were Agrawal and Srikant [3]. The general strand of knowledge-hiding research [4] has led to specific algorithms, for example, adding noise through a data swap process [5].

A critical objective area concerns individual data privacy and aims to shield the individual integrity of database records in order to prevent the re-identification of individuals or particular groups of data inference attacks. The subject of this article deals with the protection of sensitive patterns resulting from the use of data mining techniques. All privacy preservation approaches are designed to maintain data quality.

The primary representative of statistical methods [6] uses a parsimonious downgrading technique to determine if the loss of functionality associated with data not downgrading is worth further confidentiality. The reconstruction of public datasets [7,8] includes the reconstruction of non-sensitive rules produced by the algorithms C4.5 [9] and RIPPER [10]. Perturbation-based techniques involve the modification of transactions to only support non-sensitive rules [11], the removal of sensitive rules-related tuples [12], the suppression of specific attribute values [13], and the redistribution of tuples supporting sensitive patterns to maintain the ordering of the rules [14]. Another useful approach is machine learning classification over encrypted data [2,15] in which a private decision tree classifier enables the server to traverse a binary decision tree using the client’s input x such that the server does not learn the input x and the tree structure and thresholds at each node are not learned by the client. A recent work [16] proposes privacy-preserving decision tree evaluation protocols that hide the critical inputs from the counterparty using additively homomorphic encryption (AHE) like the ElGamal encryption method.

We proposed a series of techniques in our previously published works [17,18] to adequately protect against the disclosure of sensitive patterns of knowledge in classification rules mining. Without compromising the information value of the entire data set, we aim to hide sensitive rules. The class labels at the tree node corresponding to the tail of the sensitive pattern are modified after an expert selects the sensitive rules to eliminate the gain achieved by the information metric causing the split.

By preserving the class balance of every node across the sensitive path, we can assure that we will not have any change in the hierarchy order of this path due to changes in entropy of the nodes along this path. We then set the values of non-class attributes appropriately, adding new instances along the path to the root if necessary, so that non-sensitive patterns remain as unaffected as possible. This approach is critical because the sanitized data set may be subsequently published and even shared with the data set owner’s competitors, as can be the case with retail banking [19]. We extend this work in the papers [20,21] by formulating a generic look ahead technique that considers the structure of the decision tree from an affected leaf to the root. The main contribution of these publications was to improve the Swap-and-Add pass by following a look ahead approach instead of the greedy approach which was previously used. These methods can be accomplished by using linear Diophantine equations and, quite importantly, can handle in parallel any number of hiding requests by determining the overall minimum number of added instances.

The objective of the present paper is to allow the publishing or sharing of the original data set by hiding the critical rules which are produced by creating the corresponding decision tree and thus preserving to the maximum possible extent the privacy of data which have caused these critical rules to appear. In this article, we propose a novel technique which does not affect the class labels of the sensitive instances, as our previous techniques do, but instead modifies the attributes’ values of these specific instances. While the new technique may need to modify more values of the initial data set, it does so by not requiring the addition of extra instances, and it thus saves on the size of the sanitized data set. This trade-off is an extra tool in the arsenal of the data engineer who might want to explore a range of possibilities when tasked with a data hiding mandate. In the proposed method, we first identify the instances that contribute to the creation of a specific rule and then, by appropriately changing attribute values, we can successfully hide this rule with minimum impact to the rest of the decision tree.

The rest of this paper is structured in three sections. Section 2 describes the data set manipulations we employ to hide a rule while attempting to affect the decision tree minimally. In Section 3, we present three fully scaled examples of how the new technique can be applied in hiding requests, and Section 4 discusses further research issues and concludes the paper.

## 2. Materials and Methods

For our research, we have chosen decision trees as we are primarily interested in techniques that apply to “comprehensible” models—and this eventually leads to rules, trees, and other graphic models. The interpretability of rules and trees, however, has to do with how widespread they are and with the scope to associate metric quality masking in terms of both verboseness and the impact on understanding and concealment accuracy. 

One of the key advantages for decision-making tree analysis is the fact that it can assign specific values to each decision, which ultimately reduces the ambiguity of decision-making. Each possible decision scenario is shown through a clear fork and node, which makes it possible to see all possible solutions in one view. A decision tree also allows data to be divided much deeper into other decision-makers, like logistic regression, which cannot be easily achieved. The decision tree illustrates the problem in a simple and easily comprehensible format which does not require any graphical or alternative explanations. Decision trees divide data into rules-based illustrations that are easily understood. Pure entropy-based mathematics can easily replicate the reasons behind a decision tree’s rules.

**Figure 1:**
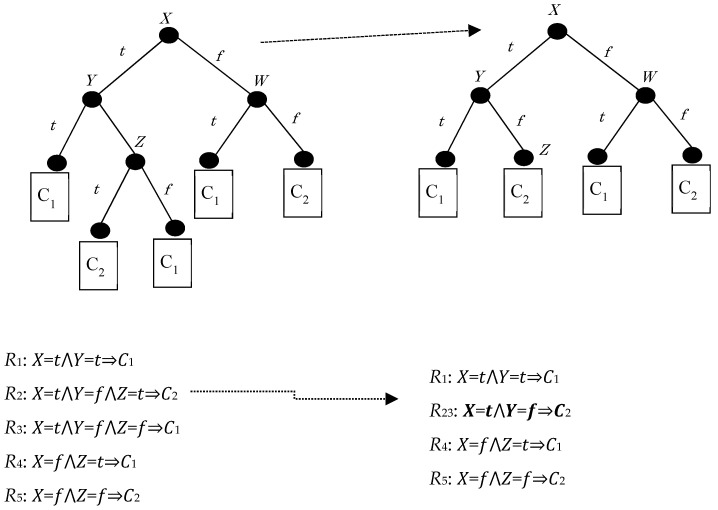
A binary decision tree before (left) and after (right) hiding and the associated rule sets.

Figure 1 below shows a baseline problem assuming the representation of a binary decision tree with binary, symbolic attributes (X, Y, and Z) and binary classes (C1 and C2). Hiding R3 implies the suppression of splitting in node Z, hiding R2 as well.

Concerning the following figure (Figure 1), hiding a rule is equivalent to hiding the leaf that corresponds to that rule (after all, a leaf corresponds to a conjunction of attribute tests which ends up in a class label). A straightforward approach to this hiding operation is the elimination of the last attribute test, which corresponds to the target leaf being merged with its sibling and their parent being turned into a leaf itself, thus decreasing the length of the original rule by one conjunction. Thus, the new terminal internal node is expected to be the parent of the target leaf; with the weaker version of our hiding technique, we merely strive to eliminate its original attribute test, and with the stronger version of our technique we aim to maximize the possibility that the new rule will be indeed shorter.

A first method of hiding R3 is to remove from the training data all instances of the leaf corresponding to R3 and to retrain the tree from the resulting dataset. This may, however, lead to a substantial restructuring of the decision tree, affecting other parts of the tree as well. 

Another approach would be to turn the direct parent of the R3 leaf into a new leaf. However, the actual dataset would not be changed. This allows an opponent to recover the original tree. 

To achieve hiding by minimally modifying the original data set, we can interpret “minimal” changes in data sets or whether the sanitized decision tree generated through hiding is syntactically close to the original with minimum modification of the initial data set. The minimum measurement of how decision-making trees are changed has been examined in the context of heuristics to ensure or approximate the effect of changes [22,23,24]. In our examples, we use the measure of the kappa statistic to compare the efficiency of the deduced decision tree after the proposed modification with the original one.

The information gain metric is used to select the test attribute at each node of the decision tree. The decision tree induction algorithm ID3 [25] used as a splitting criterion, the information gain, and its successor C4.5 use an improvement of information gain known as the gain ratio.

The split information value represents the potential information generated by splitting the training data set T into n partitions $T_{i}$, corresponding to n outcomes on attribute A.

(1) $$SplitInfo_{A}\left(T\right)=-\overset{n}{\underset{i=1}{\sum }}\frac{\left|T_{i}\right|}{\left|T\right|}\times {log}_{2}\left(\frac{\left|T_{i}\right|}{\left|T\right|}\right)$$

The gain ratio is defined as

(2) $$GainRatio\left(A,T\right)=\frac{Gain\left(A,T\right)}{SplitInfo_{A}\left(T\right)}$$

where

(3) Gain(A,T)=Info(T)−Info(A,T)

The attribute with the highest gain ratio is selected as the splitting attribute. Therefore, if we would like to suppress a particular attribute test at a node, a rule-of-thumb approach would be to try to modify the values (for that attribute) of the instances which would arrive at that node. By this modification, the resulting information gain due to that attribute would be reduced, becoming equal to zero if possible.

**Figure 2:**
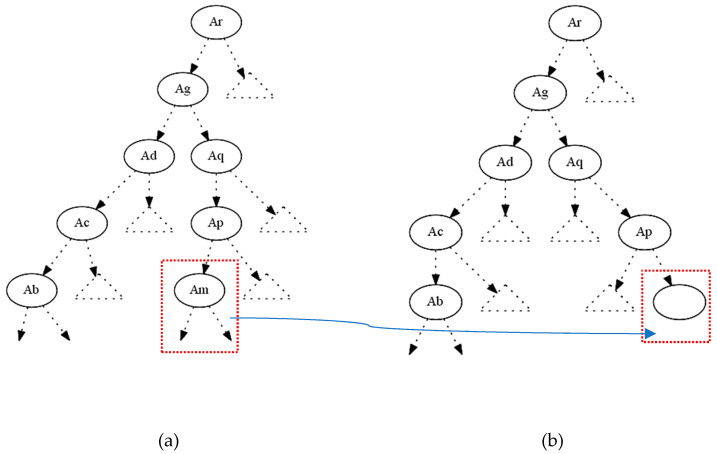
(**a**) Original decision tree; (**b**) modified decision tree with the absence of $A_{m}.$

In the figure above (Figure 2a), if we want to hide the terminal internal node $A_{m}$, we try to decrease its gain ratio at this particular splitting. The parent of the node $A_{m}$ is $A_{p}$, and the critical moment to measure the gain ratio is when the splitting test is performed to decide which node will be under the true or false value of the node $A_{p}$. The decrease of the gain ratio of $A_{m}$ could be accomplished by changing its attribute values only in the instances that correspond to this particular path (from the root $A_{r}$ until the terminal internal node $A_{m}$). If we decrease the gain ratio of $A_{m}$ and ideally became equal to zero, then it will not be displayed in its initial position. One can say that since the node $A_{m}$ is not displayed any more at this position the hiding has been successfully done.

However, we have accomplished only half of our target since other nodes (attributes) could take the $A_{m}$ position in this part of the decision tree. For that reason, we calculate for every node (attribute) the gain ratio under the conditions of the certain rule-path. Apart from the ancestors of $A_{m}$, all other attributes can be candidates to replace the initial position of $A_{m}$. Having calculated the gain ratios of all these competitors, we then proceed to decrease their gain ratios by shifting their values appropriately. We change only the attributes’ values and never their class values. This is very important since every change in a class value can affect the total entropy of the entire data set, and then every change will not only have a local impact but cause a total distortion to the whole decision tree. On the other side, a local distortion in the critical instances will only affect the entropy at this specific subdomain. By completing the procedure above we end up (ideally) with a tree which looks like the tree in Figure 2b; in other words, the final tree will be the same as the original one apart from the absence of the terminal internal node $A_{m}$.

### A Proposed Algorithm to Handle the Hiding Procedure

The algorithm LDH (Algorithm 1) locates the parent node of the leaf to be hidden and ensures that the attribute tested at that node will not generate a splitting which would allow that leaf to re-emerge. It does that by re-directing all instances following the branch from X to L towards L’s sibling by means of simply manipulating their attribute values for the attribute tested at X. If we want to pursue the stronger approach to hiding, then we extend this instance manipulation for all attributes available at X and not just the one that is being tested at X. 


**Algorithm 1 LDH**
hide (T, A, I, L, MODE)T: Tree (of at least two levels)A: Set of AttributesI: Set of InstancesL: Rule to be hidden (leaf)MODE: T/F depending on whether we want the rule’s parent to disappear begin  if L.is-leaf () then   begin    X = L.parent ()    A_x_ = X.attribute ()    I_L_ = L.instances ()    for each i in I_L_ do     i[A_x_] = not (i[A_x_])    end-do    if MODE then     begin      A_splitted_ = A − {A_w_: A_w_ appears on the path from T.root () to X)      I_X_ = X.instances()      for each a in A_splitted_ do       for each i in I_X_ do        i[a] = true       end-do      end-doendend-ifendelse// do nothing; placeholder for hiding an internal nodeend-ifend

## 3. Results

In this section, we demonstrate three examples in two different binary datasets from UCI—Machine Learning Repository [26] (SPECT and modification of CHESS). The SPECT Heart [27] training data set is based on data from cardiac Single Proton Emission Computed Tomography (SPECT) images. Each patient is classified into one of two categories: normal and abnormal. SPECT is a good data set for testing ML algorithms; it has 187 instances that are described by 23 binary attributes (A1–A22, Class). The binary values for the attributes (A1–A22) are true (t) or false (f), and the corresponding values for the Class are positive (p) or negative (n). 

The other data set, Chess End-Game [28], has 3196 instances and 36 attributes. Each instance classified is into one of two categories: win or cannot win. We modified the original data set by removing three attributes which are not described with true–false values.

The WEKA—Data Mining Software in Java [29] workbench is a collection of machine learning algorithms and data preprocessing tools. We chose for our experiments to use the classification algorithm J48 which is the implementation of the Quinlan C4.5 algorithm. C4.5 can be referred to as the statistic Classifier. This algorithm uses the gain ratio for feature selection and to construct the decision tree. It handles both continuous and discrete features. The C4.5 algorithm is widely used because of its quick classification and high precision. The C4.5 algorithm for building decision trees is implemented in Weka as a classifier called J48. The changes from the default parameter settings for this classifier were *binarySplits: True, minNumObj: 1, unpruned: True.*

**Figure 3:**
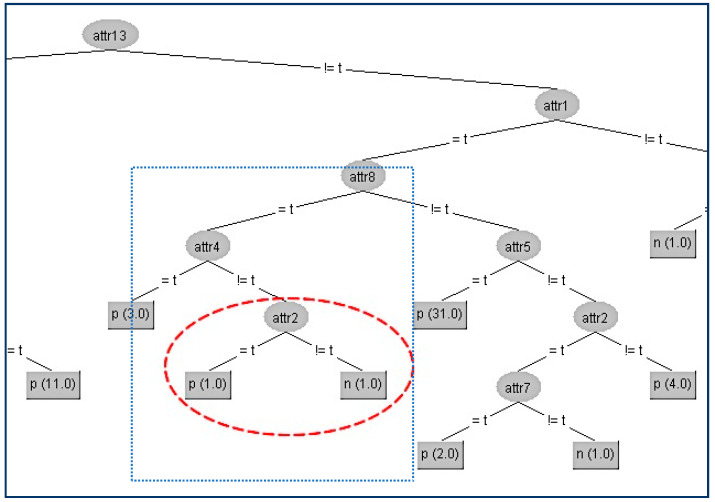
Original decision tree and Attribute 2 (A2) to be hidden.

In our first example in the SPECT data set, we try to conceal the terminal internal node (A2) which is shown in the figure below (Figure 3).

Firstly, we have to determine the instances that correspond to that node. There are two instances (#1,#2), and the original attributes’ values for these two instances are presented in the second and third column in the following table (Table 1a). With red color are denoted the modified attributes’ values. In the table (Table 1a) below are presented the gain ratios of all nodes under the false value of A4 before and after the modification (Table 1b).

In the above table, all the attributes that belong to the path from the root (A13) to the terminal internal node (A2) have been highlighted to indicate that these attributes apart from (A4) should not be changed. First, we change the attribute values of the critical node (A2) on these two instances to minimize the information gain ratio of this node. This can be achieved by swapping the value in attribute (A2) of the second instance from “t” to “f”. Apart from this change, we have also altered all other attributes’ values to decrease their gain ratio to avoid taking the place of A2. For our example, the competitor attributes are {A6, A7, A9, A10, A11, A12, A14, A16, A20}. Since there only two instances, we can match both attributes’ values to be the same to have zero gain ratios. 

**Figure 4:**
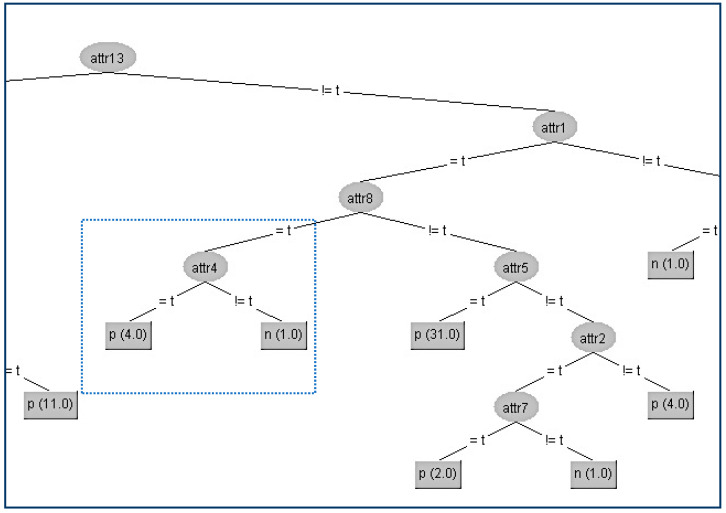
Final decision tree without Attribute 2 (A2).

In this way, we have succeeded in eliminating the contribution of the node A2 below its parent A4, and the result is shown in Figure 4.

Since the size of the above decision tree is too big to fit in one page, we present only the section of the tree around the critical node. On the website [30], the reader can find all the data set files (.arff) before and after applying our distortion method. Apart from the data sets there also exist all the decision trees in full-scale deployment as extracted from WEKA.

The kappa statistic adjusts accuracy by accounting for the possibility of a correct prediction by chance alone. Kappa values range to a maximum value of 1, which indicates perfect agreement between the model’s predictions and the true values—a rare occurrence. Values less than one indicate imperfect agreement.

Another interesting result is that the kappa statistic values which correspond to the two different trees are equal to 3 significant figures (kappa = 0.834). Also, from all other WEKA statistics, which are presented in Table 2 below, we can conclude that the node A2 has been successfully hidden without any impact on the decision tree efficiency.

The next two examples were performed using the CHESS data set since it is much more prominent in size (3196 instances) and has many more attributes (33 and the Class) than the SPECT data set. The binary values for the attributes (A1–A33) are true (t) or false (f) and the corresponding values for the Class are positive (p) or negative (n). 

**Figure 5:**
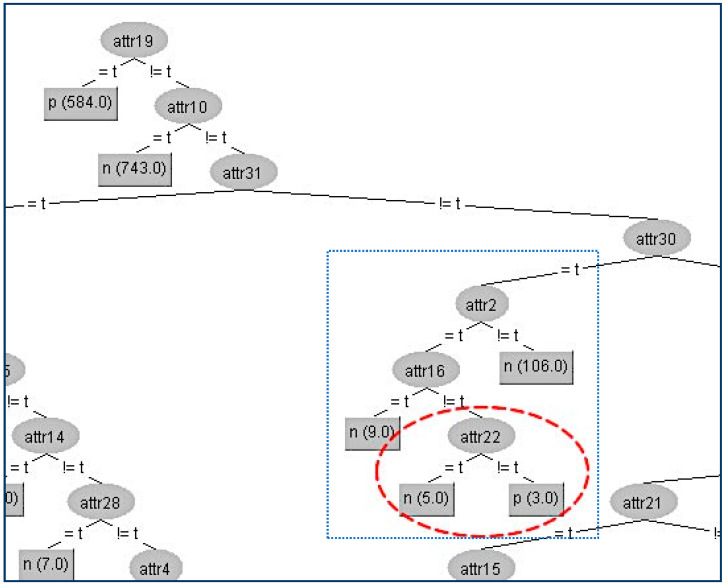
Original decision tree and the attribute (A22) to be hidden.

Next, we present the first example in the CHESS data set where we will hide the terminal internal node (A22) which is shown in Figure 5.

Firstly, we have to determine the instances that correspond to that node. In the table (Table 3a) below, all the attributes that belong to the path from the root (A19) to the terminal internal node (A22) have been highlighted to indicate that these attributes apart from the sensitive node A22 and its parent node (A16) should not be changed. As we can see in the table (Table 3a), the attribute A22 has the highest gain ratio, and this is the reason for its position in the original tree. Thus, our initial action is to decrease its gain ratio; in our example, this can be done by swapping the three “false” values into “true” ones where the class values are “positive”, which results in the gain ratio becoming zero. Then, we have to examine all other attributes’ values in order to decrease (if it is needed) their gain ratios to avoid them taking the place of A22. For our example, the competitor attributes are {A1, A14, A17, A10, A28, A29, A32}. At this point, it is essential to note that if we change an attribute value which is located in the critical path, in our case, A16, then this change also affects the gain ratios of all other values. This can be seen in the table (Table 3b) where there is not any modification in a certain attribute but its gain ratio has changed (A1, A2, A14, A17, A29). The modified attributes’ values for these eight instances are presented in the following table (Table 3a). With red color are denoted the modified attributes’ values. In the table (Table 3b) below are presented the gain ratios of all nodes under the false value of A16 before and after the modification. 

**Figure 6:**
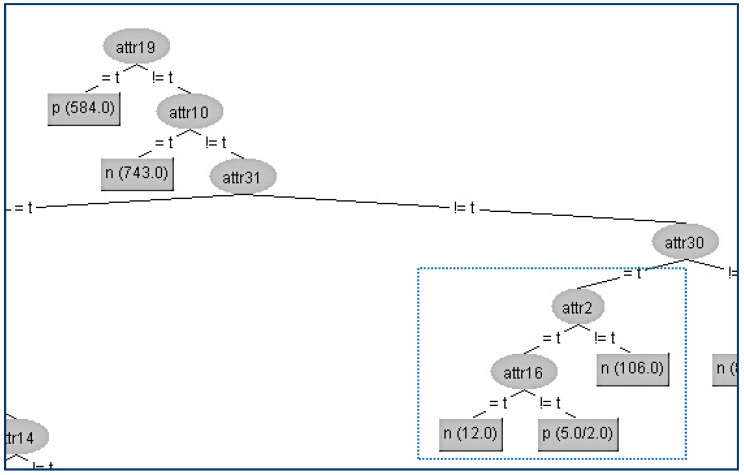
Final decision tree without Attribute 22 (A22).

In this way, we have succeeded in eliminating the contribution of the node A22 below its parent A16, and the result is shown in Figure 6.

The kappa statistic values which correspond to the two different trees are equal to 2 significant figures (kappa = 0.97). Furthermore, from all other WEKA statistics, which are presented in Table 4 below, we can conclude that the node A22 has been successfully hidden with a very limited impact on the decision tree efficiency.

**Figure 7:**
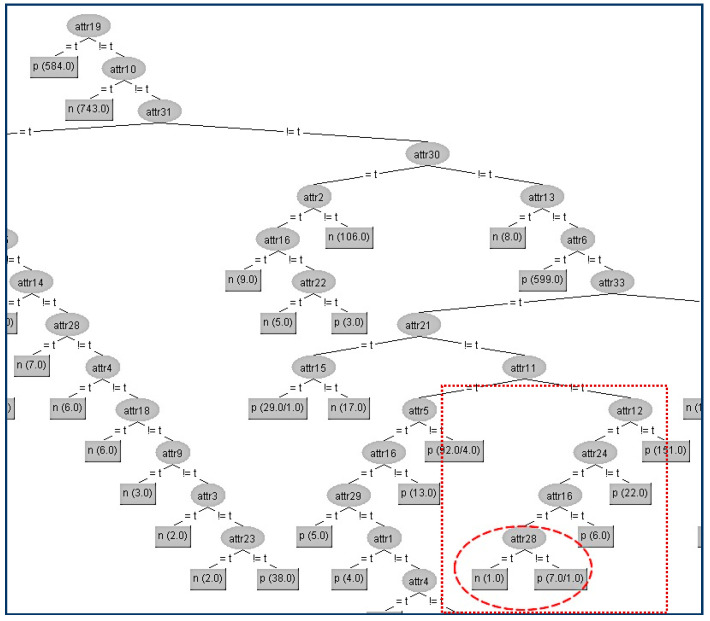
Original decision tree and Attribute 28 (A28) to be hidden.

Next, in the same data set (CHESS) we try to hide another terminal internal node (A28) which is in a lower level compared to A22 of the tree and is shown in Figure 7.

**Figure 8:**
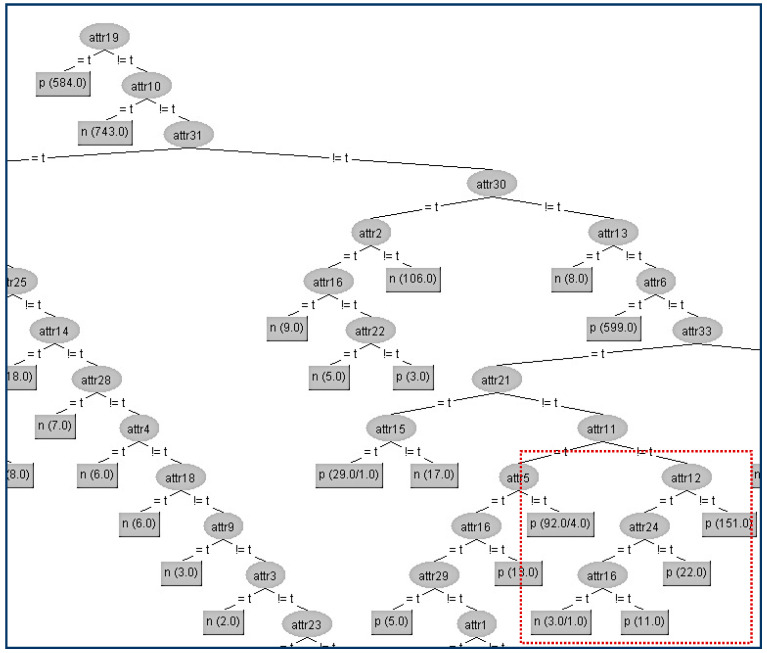
Final decision tree without Attribute 28 (A28).

Firstly, we have to determine the instances that correspond to that node. In the table (Table 5a) below, all the attributes that belong to the path from the root (A19) to the terminal internal node (A28) have been highlighted to indicate that these attributes apart from the sensitive node A28 and its parent node (A16) should not be changed. As we can see in the table (Table 5a), the attribute A28 has the highest gain ratio (except for A12, A24 which are already located in the path) and this is the reason for its position in the original tree. Thus, our initial action is to decrease its gain ratio, and in our example, this can be done by swapping the “true” value into “false” on the eighth instance, which results in its gain ratio to becoming zero. Then, we have to examine all other attributes’ values in order to decrease (if it is needed) their gain ratios to avoid them taking the place of A22. For our example, the competitor attributes are {A9, A20}. The modified attributes’ values for these eight instances are presented in the following table (Table 5a). With red color are denoted the modified attributes’ values. In the table (Table 5b) below are presented the gain ratios of all nodes under the false value of A16 before and after the modification. Next, we present the decision tree where we will hide the terminal internal node (A28) which is shown in Figure 8.

The kappa statistic values (Table 6) which correspond to the two different trees are equal to 4 significant figures (kappa = 0.9768). Thus, the node A28 has been successfully hidden without any impact on the decision tree efficiency. In this particular hiding, we observe a tiny improvement (all the error metrics are lower in the modified tree).

## 4. Discussion and Conclusions

Our new methodology allows one to specify which decision tree leaves should be hidden and then judiciously change some attribute values in specific instances in the original data set. Consequently, the next time one tries to build the tree, the to-be-hidden nodes will have disappeared as the instances corresponding to those nodes will have been absorbed by neighboring ones.

The detailed results presented above have demonstrated that the proposed method successfully hid the terminal internal nodes which were selected in two different data sets. In addition, using the proposed technique we demonstrated that in all three examples, no change occurred in the structure of the modified decision trees (when compared to the original ones) apart from the hidden node.

By using the technique that we propose in this paper, instead of the previously used look ahead approach using Linear Diophantine equations, there are two significant advantages. The first one is that we do not need to add new instances to the original data set, and the second is that our new heuristic can be performed in only one step with much lower computational complexity compared to solving systems of Linear Diophantine Equations. However, our previous published techniques [20,21] guarantee the preservation of entropy values in every node of the tree before and after the modification. 

The medium-term development goal is to have the proposed technique implemented as a standard data engineering service to accommodate hiding requests, coupled with an appropriate environment where one could specify the importance of each hiding request. In terms of research and development aspects, the most pressing questions are related to the technique’s ability to handle multivalued (also, numeric) attributes and multi-class trees. Additionally, in terms of application, we will actively investigate the options to apply our technique to a real-world problem (for example, as in reference [2]), but we must also acknowledge the substantial non-disclosure agreements which may have to accompany such real-world tests. Still, we believe that the apparent rise in interest for privacy-preserving solutions suggests that systems with a theoretical backing can be expected to appear increasingly often.

## Figures and Tables

**Table 1:** (**a**) Original and modified attributes’ values; (**b**) original and modified gain ratios.

(a)					(b)		
	#1	#2	#1	#2	Attribute	Original Gain Ratio	Modified Gain Ratio
A1	t	t	t	t	A1	0	0
A2	f	t	f	**f**	A2	1	0
A3	t	t	t	t	A3	0	0
A4	f	f	f	f	A4	0	0
A5	t	t	t	t	A5	0	0
A6	f	t	f	**f**	A6	1	0
A7	f	t	f	**f**	A7	1	0
A8	t	t	t	t	A8	0	0
A9	t	f	**f**	f	A9	1	0
A10	f	t	f	**f**	A10	1	0
A11	f	t	f	**f**	A11	1	0
A12	f	t	f	**f**	A12	1	0
A13	f	f	f	f	A13	0	0
A14	t	f	**f**	f	A14	1	0
A15	f	f	f	f	A15	0	0
A16	t	f	**f**	f	A16	1	0
A17	f	f	f	f	A17	0	0
A18	f	f	f	f	A18	0	0
A19	f	f	f	f	A19	0	0
A20	f	t	f	**f**	A20	1	0
A21	f	f	f	f	A21	0	0
A22	f	f	f	f	A22	0	0
Class	n	p	n	p			

**Table 2:** Weka output for the original and modified data sets.

	Original	Modified
Correctly Classified Instances	182	182
Incorrectly Classified Instances	5	5
Kappa statistic	0.834	0.834
Mean absolute error	0.0352	0.0352
Root-mean-squared error	0.1328	0.1328
Relative absolute error	29.2968%	29.2968%
Root relative squared error	48.8664%	48.8664%

**Table 3:** (**a**) Modified attributes’ values; (**b**) original and modified gain ratios.

(a)									(b)		
	#1	#2	#3	#4	#5	#6	#7	#8	Attr	Original Gain Ratio	Modified Gain Ratio
A1	f	f	t	f	f	f	t	t	A1	0.003383098	0.020571
A2	t	t	t	t	t	t	t	t	A2	0.813346207	1.110375
A3	f	f	f	f	f	f	f	f	A3	0	0
A4	f	f	f	f	f	f	f	f	A4	0	0
A5	f	f	f	f	f	f	f	f	A5	0	0
A6	f	f	f	f	f	f	f	f	A6	0	0
A7	f	f	f	f	f	f	f	f	A7	0	0
A8	f	f	f	f	f	f	f	f	A8	0	0
A9	f	f	f	f	f	f	f	f	A9	0	0
A10	f	f	f	f	f	f	f	f	A10	0	0
A11	f	f	f	f	f	f	f	f	A11	0	0
A12	f	f	f	f	f	f	f	f	A12	0	0
A13	f	f	f	f	f	f	f	f	A13	0	0
A14	f	f	f	f	t	f	f	f	A14	0.169914322	0
A15	f	f	f	f	f	f	f	f	A15	0	0
A16	f	f	f	f	**t**	**t**	**t**	f	A16	0.506553684	0.784201
A17	f	f	f	f	f	t	f	f	A17	0.169914322	0
A18	f	f	f	f	f	f	f	f	A18	0	0
A19	f	f	f	f	f	f	f	f	A19	0	0
A20	f	f	f	f	f	f	f	f	A20	0	0
A21	f	f	f	f	f	f	f	f	A21	0	0
A22	**t**	**t**	**t**	t	t	t	t	t	A22	1	0
A23	f	f	f	f	f	f	f	f	A23	0	0
A24	t	t	t	t	t	t	t	t	A24	0	0
A25	f	f	f	f	f	f	f	f	A25	0	0
A26	f	f	f	f	f	f	f	f	A26	0	0
A27	f	f	f	f	f	f	f	f	A27	0	0
A28	f	**t**	**t**	f	f	t	f	t	A28	0.251990035	0.020571
A29	t	t	f	t	t	t	f	f	A29	0.003383098	0.020571
A30	t	t	t	t	t	t	t	t	A30	0	0
A31	f	f	f	f	f	f	f	f	A31	0	0
A32	t	f	f	**t**	t	f	f	f	A32	0.019367128	0.020571
A33	f	f	f	f	f	f	f	f	A33	0	0
Class	p	p	p	n	n	n	n	n			

**Table 4:** Weka output for the original and modified data sets.

	Original	Modified
Correctly Classified Instances	3159 (98.8423%)	3157 (98.7797%)
Incorrectly Classified Instances	37 (1.1577%)	39 (1.2203%)
Kappa statistic	0.9768	0.9755
Mean absolute error	0.0167	0.0175
Root-mean-squared error	0.0914	0.0934
Relative absolute error	3.3492%	3.4997%
Root relative squared error	18.3009%	18.7075%

**Table 5:** (**a**) Original and modified attributes’ values; (**b**) original and modified gain ratios.

(a)										(b)		
	#1	#2	#3	#4	#5	#6	#7	#8		Attr	Original Gain Ratio	Modified Gain Ratio
A1	f	f	f	f	f	f	f	f	A1	A1	0	0
A2	f	f	f	f	f	f	f	f	A2	A2	0	0
A3	f	f	f	f	f	f	f	f	A3	A3	0	0
A4	f	f	f	f	f	f	f	f	A4	A4	0	0
A5	f	f	f	f	f	f	f	f	A5	A5	0	0
A6	f	f	f	f	f	f	f	f	A6	A6	0	0
A7	f	f	f	f	f	f	f	f	A7	A7	0	0
A8	f	f	f	f	f	f	f	f	A8	A8	0	0
A9	f	f	t	f	t	f	f	f	A9	A9	0.15107	0
A10	f	f	f	f	f	f	f	f	A10	A10	0	0
A11	f	f	f	f	f	f	f	f	A11	A11	0.26173	0.45915
A12	t	t	t	t	t	t	t	t	A12	A12	0.98794	2.21025
A13	f	f	f	f	f	f	f	f	A13	A13	0	0
A14	f	f	f	f	f	f	f	f	A14	A14	0	0
A15	f	f	f	f	f	f	f	f	A15	A15	0	0
A16	t	**f**	**f**	**f**	**f**	**f**	t	t	A16	A16	0.3529	0.96254
A17	f	f	f	f	f	f	f	f	A17	A17	0	0
A18	f	f	f	f	f	f	f	f	A18	A18	0	0
A19	f	f	f	f	f	f	f	f	A19	A19	0	0
A20	f	f	t	f	t	f	f	f	A20	A20	0.15107	0
A21	f	f	f	f	f	f	f	f	A21	A21	0	0
A22	f	f	f	f	f	f	t	t	A22	A22	0.09092	0.27402
A23	f	f	f	f	f	f	f	f	A23	A23	0	0
A24	t	t	t	t	t	t	t	t	A24	A24	0.63072	1.33031
A25	f	f	f	f	f	f	f	f	A25	A25	0	0
A26	f	f	f	f	f	f	f	f	A26	A26	0	0
A27	f	f	f	f	f	f	f	f	A27	A27	0	0
A28	f	f	f	f	f	f	f	**f**	A28	A28	0.54007	0
A29	f	f	f	f	f	f	f	f	A29	A29	0	0
A30	f	f	f	f	f	f	f	f	A30	A30	0	0
A31	f	f	f	f	f	f	f	f	A31	A31	0	0
A32	t	t	t	t	t	t	t	t	A32	A32	0	0
A33	t	t	t	t	t	t	t	t	A33	A33	0	0
Class	n	p	p	p	p	p	p	n				

**Table 6:** Weka output for the original and modified data sets.

	Original	Modified
Correctly Classified Instances	3159 (98.8423%)	3159 (98.8423%)
Incorrectly Classified Instances	37 (1.1577%)	37 (1.1577%)
Kappa statistic	0.9768	0.9768
Mean absolute error	0.0167	0.0166
Root-mean-squared error	0.0914	0.0911
Relative absolute error	3.3492%	3.3253%
Root relative squared error	18.3009%	18.2355%
